# Emerging Role of D-Amino Acid Metabolism in the Innate Defense

**DOI:** 10.3389/fmicb.2018.00933

**Published:** 2018-05-09

**Authors:** Jumpei Sasabe, Masataka Suzuki

**Affiliations:** Department of Pharmacology, School of Medicine, Keio University, Tokyo, Japan

**Keywords:** D-amino acid, D-amino acid oxidase, hydrogen peroxide, mucosal immunity, innate immunity, small intestine, neutrophil, host–microbe interaction

## Abstract

Mammalian innate and adaptive immune systems use the pattern recognition receptors, such as toll-like receptors, to detect conserved bacterial and viral components. Bacteria synthesize diverse D-amino acids while eukaryotes and archaea generally produce two D-amino acids, raising the possibility that many of bacterial D-amino acids are bacteria-specific metabolites. Although D-amino acids have not been identified to bind to any known pattern recognition receptors, D-amino acids are enantioselectively recognized by some other receptors and enzymes including a flavoenzyme D-amino acid oxidase (DAO) in mammals. At host–microbe interfaces in the neutrophils and intestinal mucosa, DAO catalyzes oxidation of bacterial D-amino acids, such as D-alanine, and generates H_2_O_2_, which is linked to antimicrobial activity. Intestinal DAO also modifies the composition of microbiota through modulation of growth for some bacteria that are dependent on host nutrition. Furthermore, regulation and recognition of D-amino acids in mammals have additional meanings at various host–microbe interfaces; D-phenylalanine and D-tryptophan regulate chemotaxis of neutrophils through a G-coupled protein receptor, D-serine has a bacteriostatic role in the urinary tract, D-phenylalanine and D-leucine inhibit innate immunity through the sweet taste receptor in the upper airway, and D-tryptophan modulates immune tolerance in the lower airway. This mini-review highlights recent evidence supporting the hypothesis that D-amino acids are utilized as inter-kingdom communication at host–microbe interface to modulate bacterial colonization and host defense.

## Introduction

**FIGURE 1:**
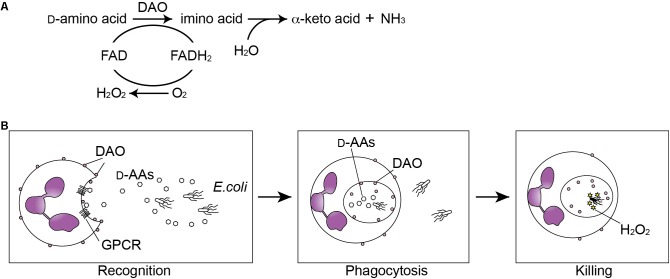
Oxidation of D-amino acids by D-amino acid oxidase (DAO) generates anti-microbial H_2_O_2_. **(A)** DAO catalizes oxidative deamination of D-amino acids. The D-amino acid is oxidized to an imino acid with reduction of FAD to FADH_2_, which is subsequently oxidized to FAD with reduction of oxygen into hydrogen peroxide. The imino acid is then non-enzymatically hydrolyzed to the corresponding alpha-keto acid and ammonia. **(B)** Neutrophils recognize bacterial D-amino acids through the G protein-coupled receptor (GPCR) GPR109B and are chemoattracted by bacteria. DAO oxidizes bacterial D-amino acids during the phagocytosis and generated H_2_O_2_ kills the bacteria.

Among all domains of life, bacteria have the largest capacity to produce wide variety of D-amino acids, whereas archaea and eukaryotes are thought to synthesize generally two kinds of D-amino acids, D-serine and D-aspartate. Bacteria utilize diverse D-amino acids in multiple biological processes to support their growth, to regulate spore germination, and to configure or remodel their cell wall (Cava et al., 2011). By contrast, mammals utilize D-serine in neurophysiology and D-aspartate in neurogenesis and endocrine systems (Fujii and Saito, 2004). Metabolism of D-amino acids in mammals involves two flavoenzymes: D-amino acid oxidase (DAO) and D-aspartate oxidase. DAO catalyzes stereoselective oxidative deamination of multiple neutral and basic D-amino acids, which yields alpha-keto acids, ammonium ion, and hydrogen peroxide (**Figure 1A**, showing the exact chemical reaction). DAO is rarely found in bacteria, but occurs widely in most eukaryotes from yeast to humans with the exception of plants (Pollegioni et al., 2007). DAO activity was first described in the porcine kidney by Krebs (1935), but its physiological role was not clear because its substrates had been regarded as “unnatural” isomers of amino acids. After discovery of D-amino acids as integral components of bacterial cell wall in 1950s, Cline and Lehrer in 1969 identified DAO activity in granule fraction of human neutrophilic leukocytes (Cline and Lehrer, 1969), which is linked to bactericidal activity of leukocytes by H_2_O_2_ produced through oxidation of bacterial D-amino acids (Eckstein et al., 1971; DeChatelet et al., 1972). DAO has received attention by neuroscientists for past few decades because DAO in the mammalian hindbrain degrades its physiological endogenous substrate D-serine, which binds to *N*-methyl D-aspartate (NMDA) glutamate receptors and plays crucial roles in neurophysiology and pathology (Mothet et al., 2000; Shleper et al., 2005; Sasabe et al., 2007; Basu et al., 2009; Mitchell et al., 2010; Mustafa et al., 2010; Balu et al., 2013). More recently, DAO was identified in epithelial surface of the mammalian small intestine, where interplay between mammalian DAO and bacterial D-amino acids modifies commensal bacteria and mucosal defense (Sasabe et al., 2016). Notably no mammalian genes have homology to any known bacterial genes encoding synthetic enzymes for D-amino acids, and many of bacterial D-amino acids are thought bacteria-specific metabolites. Therefore, metabolism of bacterial D-amino acids by mammalian DAO or other molecules in the host–microbial interface may serve as a type of bacterial recognition.

## Distribution of DAO Substrates in Mammals

D-amino acid oxidase is distributed to the proximal tubules in the kidney with the highest expression, hepatocytes in the liver (exceptionally not detectable in mice), astrocytes in the hindbrain of the central nervous system, neutrophils, and epithelium of the small intestine (Pollegioni et al., 2007; Koga et al., 2017). Although DAO has oxidative activity selective to D-enantiomers of amino acids, DAO has a broad spectrum of substrates including multiple neutral and basic D-amino acids. In fact, mice lacking systemic DAO activity due to a missense mutation of G181R (DAO-null mice) show increased levels of various D-amino acids, including D-alanine, D-leucine, D-methionine, D-proline, D-phenylalanine, D-serine, and D-tyrosine, in the multiple tissues and body fluids (Koga et al., 2017). Since bacteria have the largest genetic capacity to produce wide variety of D-amino acids, most of the DAO substrates except for D-serine have been regarded to originate from bacteria. Among those D-amino acids, D-alanine and D-serine are considered major substrates of DAO in mammals because the two D-amino acids show marked increases compared to other D-amino acids in the DAO-null mice (Miyoshi et al., 2011; Koga et al., 2017).

The D-alanine and D-serine have different origins in mammals. D-Serine is produced in mammals through conversion from L-serine by an endogenous enzyme, serine racemase (SR) (Wolosker et al., 1999), expressed primarily in neurons of the central nervous system (Miya et al., 2008). The concentration of D-serine is highest (10–30% of total serine) at submillimolar level in the forebrain (Hashimoto et al., 1992; Miyoshi et al., 2009; Suzuki et al., 2017), where D-serine serves as an endogenous coagonist with L-glutamate to activate NMDA receptors (Mothet et al., 2000; Basu et al., 2009). By contrast, DAO degrades D-serine and retains it at a low level (less than 1% of total serine) in the hindbrain and spinal cord (Miyoshi et al., 2009). In the periphery, D-serine level is usually low with the exception of that in the urine (Miyoshi et al., 2009). The D-serine is over 50% of total urinary serine and is one of the most abundant amino acids in mammalian urine, where D-serine has a bacteriostatic role against uropathogenic bacteria (Roos and Klemm, 2006; Korte-Berwanger et al., 2013) by inhibiting L-serine metabolism and synthesis of pantothenate (Cosloy and McFall, 1973) and by modulating virulence gene expression (Roesch et al., 2003; Anfora and Welch, 2006; Anfora et al., 2007). It is also known that mammalian D-serine can repress efficient colonization of enterohaemorrhagic *Escherichia coli* (EHEC) by selectively inhibiting expression of a type III secretion system, which allows intimate attachment of EHEC to the host cells (Connolly et al., 2015). Such anti-bacterial D-serine is rarely synthesized in bacteria with exception of vancomycin-resistant *Enterococci* (Sieradzki and Tomasz, 1996; De Jonge et al., 2002; Reynolds and Courvalin, 2005) and therefore, bacteria have been thought to develop sensing system of host D-serine to recognize niche and colonize to favorable sites within the host (Connolly et al., 2016).

Another major substrate of DAO in mammals is bacterial D-alanine, one of most common D-amino acids present in the bacterial cell wall. While mammals are not capable of synthesizing D-alanine, most bacteria encode two different PLP-dependent alanine racemases, DadX and Alr, which are established drug targets for antibiotics. Both racemases catalyze the same reaction, but are components of distinct molecular pathways. In contrast to association of DadX with L-alanine catabolism (D-alanine is subsequently converted into pyruvate), Alr synthesizes the D-alanine that is utilized for peptidoglycan synthesis (Walsh, 1989; Watanabe et al., 2002). The D-alanine is incorporated into the peptides to cross-linking repeated disaccharide and provides chemical resistance to most known proteases (Nagata et al., 1998). It has become clear that intestinal D-alanine as well as other D-amino acids including D-proline, and D-glutamate, is produced exclusively by intestinal microbiota (Sasabe et al., 2016). Furthermore, Konno et al. (1990, 1993) have shown using antibiotic-treated and germ-free (GF) mice that vast majority of D-alanine in the serum and urine is of bacterial origin. Therefore, D-alanine produced by intestinal microbiota is, at least in part, uptaken in the intestine, circulated, and excreted into the urine. Mechanisms regulating *in vivo* D-alanine kinetics, such as D-alanine transport and metabolism, still remain largely unclear.

## Role of DAO in the Innate Defense by Leukocytes

D-amino acid oxidase, conserved widely in eukaryotes but not in bacteria (Pollegioni et al., 2007), is able to generate H_2_O_2_ through catabolism of D-amino acids (usually of bacterial origin), and therefore, DAO has classically been considered a potential component of the innate defense in mammals. Cline and Lehrer (1969) first identified DAO activity in granule fraction of guinea pig and human neutrophilic leukocytes, which is linked to bactericidal activity of leukocytes (Eckstein et al., 1971; DeChatelet et al., 1972). They have also shown *in vitro* that oxidation of bacterial metabolites such as D-amino acids by DAO generates H_2_O_2_ and subsequently activates chloride ions together with myeloperoxidase to kill *E. coli*. A later study using electron microscope showed that DAO is localized to the neutrophilic surface, internalized during phagocytosis, and is able to produce H_2_O_2_ within the phagosome (Robinson et al., 1978). On the other hand, *in vivo* bactericidal effect of neutrophilic DAO remains controversial. Neutrophils obtained from patients with chronic granulomatous disease (CGD), which is a primary immunodeficiency that interferes production of reactive oxygen species by phagocytes (i.e., neutrophils and macrophages) and leads to recurrent or persistent intracellular bacterial and fungal infections, have comparable DAO activity to those from control patients with bacterial infections (Eckstein et al., 1971). On the basis of the observation, it seems unlikely that oxidation of D-amino acids is the primary source of H_2_O_2_ generation during phagocytosis, where NADPH oxidase plays a major role (Nguyen et al., 2017). However, patients with CGD are rarely infected with catalase-negative organisms (Winkelstein et al., 2000), suggesting that a source of H_2_O_2_ stress independent of NADPH oxidase, such as DAO, may be selective for certain infections in these patients. It had remained unclear for more than 40 years if DAO plays a role *in vivo* against bacterial infection until the study using the DAO-null mice by Nakamura et al. (2012). The DAO-null mice were injected intravenously with *Staphylococcus aureus*, and showed increased number of the bacteria in the kidney and reduced survival rate compared to wild-type controls. However, they did not find *ex vivo* bactericidal effect of DAO in neutrophils derived from peritoneal cavity against *S. aureus*, warranting further examination using conditional knockout to unveil the physiological role of neutrophilic DAO. Another *in vivo* study has shown that neutrophil DAO functions to exert bactericidal activity on intraperitoneally injected *Salmonella typhimurium* at early stages of infection (Tuinema et al., 2014), when neutrophils are the major cell type infected by *Salmonella* (Loetscher et al., 2012). Interestingly, *S. typhimurium* limits exposure to oxidative damage elicited by DAO through importing D-alanine by dalS, an ABC importer specific for D-alanine. Indeed, dalS mutants of *S. typhimurium* are exposed to greater H_2_O_2_ stress than the wild type *in vivo*, which is attenuated by the presence of a chemical DAO inhibitor, 6-chloro-1,2-benzisoxazol-3(2H)-one (CBIO) (Tuinema et al., 2014). On the other hand, aromatic D-amino acids, such as D-phenylalanine and D-tryptophan, act as chemoattractant factors for human leukocytes through a G protein-coupled receptor, GPR109B (Irukayama-Tomobe et al., 2009). Collectively, these studies imply a mechanistic insight into a host–pathogen interaction; neutrophils are chemo-attracted by bacterial D-amino acids and kill bacteria through oxidation of the amino acids in phagosome (**Figure 1B**), while bacteria evade bactericidal activity of DAO through actively importing its substrates.

## DAO as an Anti-Microbial Factor in the Mucosal Innate Defense

**FIGURE 2:**
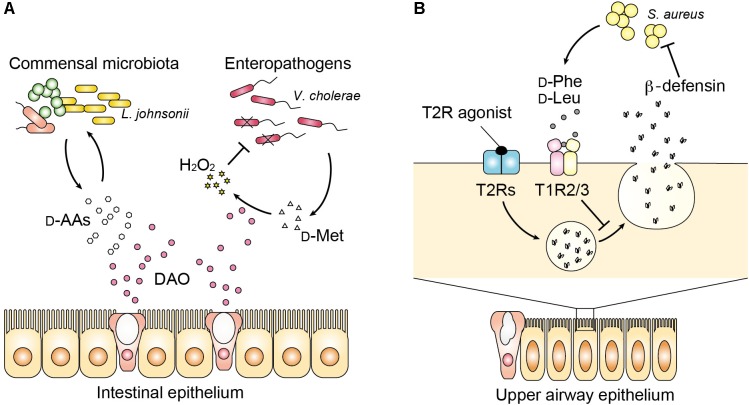
Host–microbe communication with D-amino acids in the mucosa. **(A)** DAO influences luminal bacteria in the intestinal mucosa with bimodal functions. DAO modifies the composition of commensal microbiota partly by modulating availability of D-amino acids for bacterial growth. On the other hand, DAO limits colonization of enteropathogens such as *Vibrio cholerae* by generation of H_2_O_2_ through oxidation of bacterial D-amino acids. **(B)** In the upper airway, bacterial D-phenylalanine and D-leucine bind to the sweet taste receptor (T1R2/3). Release of antimicrobial peptides including beta-defensin by activation of the bitter taste receptor (T2Rs) is suppressed by signaling from the sweet taste receptor.

In addition to the bactericidal function of neutrophilic DAO, regulation of D-amino acids by DAO has recently been associated with mucosal homeostasis (Sasabe et al., 2016). DAO activity was reported in luminal epithelium of the small intestine in fish (Sarower et al., 2003a,b), chickens (Brachet and Puigserver, 1992), mice and humans (Sasabe et al., 2016). Intestinal DAO was described in common carp as a metabolizing agent for D-alanine because free D-alanine is abundant exceptionally in aquatic invertebrates, besides bacteria, such as crustaceans and bivalve mollusks (D’Aniello and Giuditta, 1980; Matsushima et al., 1984), which are potential food sources of these fish. Of note, feeding carps with D-alanine increases intestinal DAO activity by eightfold, suggesting that inducible nature of fish DAO is associated with its role in metabolizing exogenous D-alanine (Sarower et al., 2003b). Distribution of intestinal DAO in chickens shows similar pattern as that in mice. In chicken and mice, DAO activity is detected in the mucosa of small intestine and higher in the proximal part compared to the distal part within small intestine (Brachet and Puigserver, 1992; Sasabe et al., 2016). Importantly, intestinal DAO in mice and humans localizes to Muc2-positive secretory vesicles of goblet cells as well as to enterocytes, which is inducible by the presence of vancomycin-sensitive intestinal microbiota, and oxidizes DAO substrates such as D-alanine in the mucosa and epithelium (Sasabe et al., 2016). Therefore, host DAO is induced and released in response to certain intestinal microbiota to react to microbial metabolites. Such microbe–host interaction can affect intestinal microbiota presumably in two ways (Sasabe et al., 2016) (**Figure 2A**). The first is the similar way as described in the leukocytes that oxidation of bacterial D-amino acids generates bactericidal H_2_O_2_, which limits colonization of pathogenic bacteria including *Vibrio cholerae* in the small intestine. *V. cholerae* and *V. parahaemolyticus* are more sensitive to DAO than *Listeria monocytogenes*, EHEC, or *Salmonella enterica* (Sasabe et al., 2016), in part, because *Vibrio* produces a good substrate of DAO D-methionine by expressing a broad spectrum amino acid racemase in *Vibrio* (bsrV) (Lam et al., 2009). On the other hand, commensal bacteria *Lactobacilli* are resistant to H_2_O_2_ (Serata et al., 2012) and not killed by DAO. Therefore, bactericidal activity of DAO may be defined by bacterial production/release of DAO substrates and resistance to H_2_O_2_ stress. The second is associated with nutritional niche for certain bacteria that are dependent on host nutrients and receive benefit from D-amino acids for their growth, which modifies the composition of the commensal microbiota. For example, loss of DAO results in increase of *Lactobacillus johnsonii* (Sasabe et al., 2016), which completely lacks genes encoding biosynthetic pathways for amino acids (Pridmore et al., 2004) and obtains growth support by D-alanine (van der Kaaij et al., 2004). Furthermore, of note, loss of such mucosal homeostasis by DAO increases the level of secretory IgA in the feces (Sasabe et al., 2016). Thus, these findings identify previously unrecognized physiological role of metabolizing D-amino acid by intestinal DAO in mucosal immunity.

## Modification of Mucosal Immunity by Bacterial D-Amino Acids Beyond DAO

In the upper respiratory airway, it has become clear that some D-amino acids modify innate immunity although involvement of DAO has not been studied yet. Human saliva contains substantial amount of free D-amino acids such as D-alanine (∼30% of total alanine), D-proline (∼20% of total proline), and D-aspartate (∼10% of total aspartate) (Battistone and Burnett, 1961; Syrjanen et al., 1990; Nagata et al., 2006). The whole chiral profile of salivary amino acid remains uncertain, but more kinds of D-amino acids may exist considering their diverse origins including food and oral commensal microbiota. Whereas most L-amino acids are known to taste bitter, D-amino acids taste usually sweet presumably because of stereoselectivity of sweet taste receptors (T1R2/3), which preferentially bind the D-amino acids including D-tryptophan, D-phenylalanine, D-leucine, and D-histidine (Bassoli et al., 2014). Interestingly, sweet and bitter taste receptors present in the upper airway are known to influence antimicrobial innate immune responses. Activation of bitter taste receptors (T2Rs) stimulates surrounding epithelial cells to release antimicrobial peptides (Lee et al., 2014), but the sweet taste receptor (T1R) inhibits this response (Margolskee, 2002). Two D-amino acids (D-leucine and D-phenylalanine), found in respiratory isolates of *Staphylococcus* species inhibit the release of antimicrobial peptides by activating T1R2/3, and increase cell death of human sinonasal epithelial culture in response to infection with methicillin-resistant *S. aureus* (Lee et al., 2017) (**Figure 2B**). Thus, D-amino acids produced by nasal microbiota can inhibit innate immune response through sweet taste receptors and may shape the microbial community of the upper airways. Kepert et al. (2017) have shown further evidence to support the role of bacterial D-amino acids in the mucosal immunity both in the lower airway and intestine. They identified D-tryptophan, screened from supernatants of probiotic bacteria, to reduce secretion of chemokine ligand 17 (CCL17) in a human Hodgkin lymphoma T-cell line and to induce IL-10 and decrease LPS-induced IFN-gamma, IL-12, and IL-5 in human monocyte-derived dendritic cells. Oral supplementation of D-tryptophan in mice alters diversity of gut microbiota, increases numbers of regulatory T cells in the lung and colon, decreases lung Th2 responses, and ameliorates allergic airway inflammation and hyperresponsiveness (Kepert et al., 2017). Although further mechanisms underlying connection between innate and acquired immunity modified by bacterial D-amino acids remain largely unknown, recognition of bacterial D-amino acids by the mammalian enzyme or receptors may play a significant role in the mucosal immunity and homeostasis.

## Conclusion

In mammals, intrinsic D-serine and D-aspartate have received great attention for their neuromodulatory roles in the central nervous system. In this mini-review, we have shed light on previously less-focused D-amino acids originated from commensal or pathogenic bacteria. As bacteria produce and release a largely distinct set of D-amino acids from mammals, accumulating evidence show that bacterial D-amino acids serve as inter-kingdom signals linked to innate defense in mammals. At the host–microbe interfaces, host reacts to bacteria through enantioselective recognition of D-amino acids by DAO or sweet taste receptors, and provides direct toxic response or indirect actions through modulating antimicrobial peptides. Furthermore, such recognition of bacterial D-amino acids by host may further mediate signals to modulate adaptive immunity.

## Author Contributions

JS and MS contributed to the planning and writing of the manuscript.

## Conflict of Interest Statement

The authors declare that the research was conducted in the absence of any commercial or financial relationships that could be construed as a potential conflict of interest.
